# A randomized controlled trial of endoscopic steroid injection for prophylaxis of esophageal stenoses after extensive endoscopic submucosal dissection

**DOI:** 10.1186/s12876-014-0226-6

**Published:** 2015-01-22

**Authors:** Hiroaki Takahashi, Yoshiaki Arimura, Satoshi Okahara, Junichi Kodaira, Kaku Hokari, Hiroyuki Tsukagoshi, Yasuhisa Shinomura, Masao Hosokawa

**Affiliations:** Department of Gastroenterology, Keiyukai Daini Hospital, Hondori-13, Shiroishi-ku, Sapporo, 003-0027 Japan; Department of Gastroenterology, Rheumatology, and Clinical Immunology, Sapporo Medical University, S-1, W-16, Chuo-ku, Sapporo, 060-8543 Japan; Department of Gastroenterology, Keiyukai Sapporo Hospital, Hondori-14, Shiroishi-ku, Sapporo, 003-0027 Japan; Department of Surgery, Keiyukai Sapporo Hospital, Hondori-14, Shiroishi-ku, Sapporo, 003-0027 Japan

**Keywords:** Esophageal stenosis, Steroid, Local injection, Endoscopic submucosal dissection (ESD), Early squamous cell carcinoma of esophagus

## Abstract

**Background:**

Esophageal stenosis following endoscopic submucosal dissection (ESD) is a serious adverse event that makes subsequent management more difficult.

**Methods:**

This parallel, randomized, controlled, open-label study was designed to examine whether local steroid injection is an effective prophylactic treatment for esophageal stenoses following extensive ESD. This single center trial was conducted at the Keiyukai Hospital, a tertiary care center for gastrointestinal disease in Japan [University Hospital Medical Network Clinical Trial Registry (UMIN-CTR) on 15 September 2011 (UMIN000006327)]. Thirty-two patients with mucosal defects involving ≥75% of the esophageal circumference were randomized to receive a single dose of triamcinolone acetonide injections (n = 16) or be treated conventionally (n = 16). The primary outcome was the frequency of stricture requiring endoscopic dilatation; the surrogate primary endpoint was the number of dilatation sessions needed. Secondary outcomes included adverse event rates, the minimum diameter of the stenotic area and the duration of the course of dilatation treatments.

**Results:**

The frequency of stricture was not significantly different between the groups because of insufficient statistical power, but the number of dilatation sessions required was significantly less in the steroid group (6.1 sessions [95% confidence interval, CI 2.8–9.4] *versus* 12.5 [95% CI 7.1–17.9] sessions in the control group; *P* = 0.04). The perforation rate was similar in both groups. The minimum diameter of stenotic lumens was significantly greater in the treatment group than controls (11.0 mm *versus* 7.1 mm, respectively; *P* = 0.01). The perforation rate was not significantly different between the groups (1.0% *versus* 0.5% in the treatment and control group, respectively). Steroid injection was effective in cases of mucosal defects encompassing the entire esophageal circumference.

**Conclusions:**

Prophylactic endoscopic steroid injection appears to be a safe means of relieving the severity of esophageal stenoses following extensive ESD.

## Background

In Japan, endoscopic submucosal dissection (ESD) is widely accepted as a standard treatment for early esophageal squamous cell carcinomas without documented metastasis. The ESD technique has been shown to reduce the risk of local recurrence, and perforations arising as a consequence of treatment are generally well tolerated [1]. As ESD can excise larger lesions than endoscopic mucosal resection, it is becoming increasingly popular, but esophageal stenosis after removal of large lesions by ESD is a major concern. Mucosal defects extending over three-quarters of the circumference of the esophagus after endoscopic resection are closely associated with the subsequent development of esophageal stenosis [2], which can cause dysphagia and impair quality of life.

Patients with esophageal stenosis are frequently treated by endoscopic dilatation therapy. The risk of perforation complicating the procedure increases with the number of therapeutic sessions [3]. It is important to identify ways of preventing esophageal stenosis after ESD, and minimizing the complications associated with treatment when it does arise. Treatment options for esophageal stenosis include mechanical dilatation with a bougie or balloon, stent placement and autologous keratinocyte implantation [4]. Dilatation therapies may, however, have a higher incidence of adverse events and recurrence rates than previously thought [5-7]. Therefore, simple, safe, reliable and inexpensive approaches are needed to cope with iatrogenic esophageal stenoses.

Systemic [8] or local [9,10] administration of steroids is reported to be an effective means of addressing esophageal stenosis after ESD, as well as for peptic stenosis [11-13]. Although systemic steroids should be avoided in patients with diabetes mellitus or hypertension, locally administered steroids appear to be safe in the vast majority of patients [10]. Locally administered steroids have minimal systemic effects owing to the small dose administered and short duration of exposure [10]. Previous retrospective and controlled prospective studies of endoscopic steroid injection therapy have found that the incidence of esophageal stenosis following ESD in patients treated with steroid was 10–19%, compared with 66–75% in untreated control groups. In addition, steroid injection therapy significantly reduced the number of required dilatation sessions.

To our knowledge, no randomized studies to date have analyzed the potential preventative benefits of endoscopic steroid injection therapy, or whether it is safe and effective, for stenosis caused by a mucosal defect involving the entire circumference of the esophagus after ESD. We undertook a prospective, randomized controlled trial to analyze the prophylactic effects of endoscopic steroid injection therapy for esophageal stenoses complicating extensive ESD.

## Methods

This randomized, controlled, open-label study was performed at Keiyukai Sapporo Hospital, Japan. All participants gave their written informed consent, based on the Helsinki Declaration (1964, 1975, amended in 1983, 2003 and 2008) of the World Medical Association, and the Ethics Committee of Keiyukai Sapporo Hospital approved the study protocol. The study was designed according to the CONSORT guidelines and was registered with the University Hospital Medical Network Clinical Trial Registry (UMIN-CTR) on 15 September 2011 (UMIN000006327).

### Study groups

**Figure 1:**
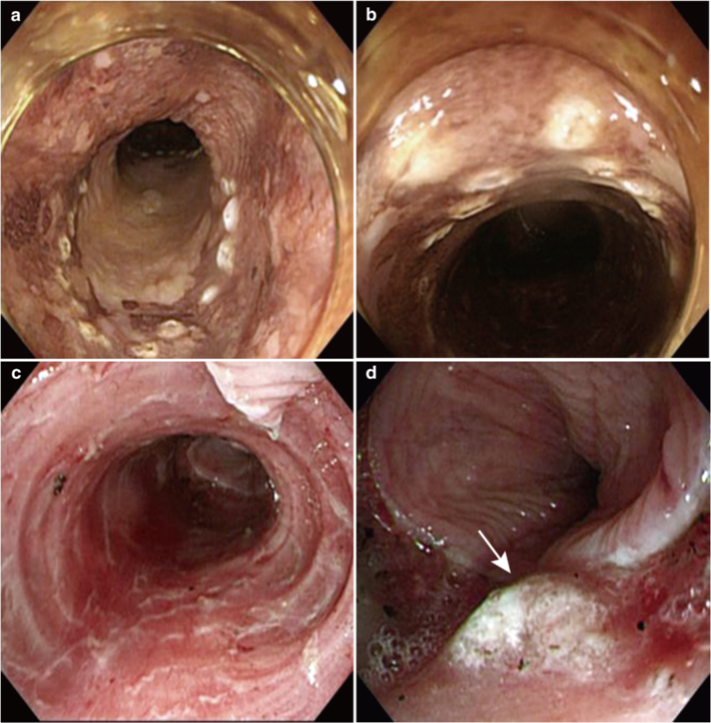
**Typical endoscopic views of the esophagus in a patient in the injection group. a**. A superficial esophageal carcinoma in the middle esophagus. The entire circumference of the lesion was marked out by electrocautery using a needle-knife at least 1 mm from the tumor border, confirmed by a Lugol-unstained region. **b**. This tumor encompasses half the circumference of the esophagus, as seen in the center of the lesion. **c**. The artificial ulcer encompassed the entire circumference after ESD. **d**. Injection of triamcinolone into the ulcer (white arrow).

Patients who had undergone ESD to treat histologically confirmed early squamous cell carcinoma of the esophagus from February 2010 to October 2011 and who were expected to have a mucosal defect encompassing ≥75% of the circumference of the esophageal mucosa after ESD were eligible for the study. Patients who received additional adjuvant treatments, such as surgery or chemoradiation therapy, and patients who were not regularly or adequately followed-up were excluded. Depth of tumor invasion was determined based on the findings of endoscopy and/or endoscopic ultrasonography. Mucosal to slightly invasive submucosal cancers (of invasion less than 200 μm in depth) were regarded as indications for ESD. Removal of a carcinoma involving two-thirds of the circumference of the esophagus by ESD was expected to result in a mucosal defect spanning more than three-quarters of the circumference. Patients enrolled in the study were randomized to receive steroid injection therapy or to be treated conventionally. Randomization was computer-generated with concealed allocation using sequentially numbered containers. Data were collated at Sapporo Medical University and independently analyzed by one author (Y.A.). The baseline demographic and clinical characteristics of the study population were compared on the basis of age, sex, tumor location, proportion of the esophageal circumference involved, number of multiple Lugol voiding lesions [14], clinical T factor [15] and follow-up period (Table 1). All ESD procedures (Figure 1A–C) were performed as described in our previous report [1]. The characteristics of the ESD procedures undertaken in the treatment and control groups were compared on the basis of the size of the tumor and resected specimen, depth of invasion, operation time, pathological margin, mucosal circumferential defect and peri-procedural perforation (Table 2).

**Table 1:** **Baseline characteristics of the study population**

	**Treatment group**	**Control group**	**p-value**
	**n = 16**	**n = 16**	
**Age, years mean ± SD (range)**	70.0 ± 9.7 (48–89)	71.0 ± 7.1 (58–83)	0.74
**Sex, male/female**	13/3	12/4	0.99
**Tumor location, n (upper/middle/lower)**	1/10/5	2/9/5	0.72
**Proportion of esophageal circumference involved, n(≥2/3 to <3/4/≥3/4 to <1/1)**	4/7/5	6/5/5	0.57
**Multiple Lugol voiding lesions, n (%)**	8 (50.0)	7 (43.8)	0.99
**Clinical T factor, n (m1/m2/m3/sm1)**	1/12/3/0	3/8/4/1	0.40
**Follow-up period, month mean ± SD (range)**	16.1 ± 5.6 (10–27)	16.9 ± 5.4 (9–27)	0.66

Abbreviation: SD, standard deviation.

**Table 2:** **Characteristics of endoscopic submucosal dissection procedures**

	**Treatment group**	**Control group**	**p-value**
	**n = 16**	**n = 16**	
**Size of carcinoma, mm mean ± SD (range)**	58 ± 16 (28–92)	53 ± 19 (30–90)	0.40
**Size of resected specimen, mm mean ± SD (range)**	68 ± 14 (43–97)	62 ± 17 (39–101)	0.29
Depth of invasion, n (m1/m2/m3/sm1/sm2*)	2/11/2/0/1	2/6/6/0/2	0.35
**Operation time, min mean ± SD (range)**	89.6 ± 37.5 (36–176)	88.3 ± 44.5 (44–235)	0.93
**Pathological margin free, n (%)**	15 (93.8)	15 (93.8)	0.99
**Mucosal defect of circumference, n (<75% / ≥75%)**	11/5	11/5	0.99
**Perforation by ESD, n (%)**	0/16 (0)	0/16 (0)	0.55

Abbreviations: m1, carcinoma *in situ*; m2, intramucosal invasive carcinoma limited to the lamina propria mucosa; m3, carcinoma limited to the muscularis mucosa; sm2, submucosal invasion between sm1 (slight invasion less than 200 μm in depth) and sm3 (massive invasion); SD, standard deviation; ESD, endoscopic submucosal dissection.

*Three patients with sm2 invasion had relative contraindications for surgical intervention for one or more of the following reasons: age considerations (the patients were 76, 79 and 89 years old), serious medical conditions (cerebral vascular disease, multiple primary cancers, low performance status), and/or patients’ decisions to decline surgery. A treatment plan for these patients was carefully chosen under full informed consent.

### Protocol for endoscopic steroid injection and dilatation therapy

As previously described [9,10], patients who did not develop immediate complications, such as perforation or bleeding, during the ESD procedure and who had been allocated to the steroid injection group were endoscopically injected with triamcinolone acetonide (Kenacort-A®, 40 mg/ml, Bristol-Myers Squibb, Anagni, Italy) immediately after the procedure. Triamcinolone was diluted with 0.9% NaCl to a final concentration of 10 mg/ml, then 0.5 ml aliquots were injected at the base of the artificial ulcer using a 25-gauge, 3 mm needle (TOP Corporation, Tokyo, Japan; Figure 1D). Injection commenced at the distal edge of the ulcer base and was repeated evenly at points 10 mm apart towards the proximal edge, taking care to avoid injuring the muscularis propria.

**Figure 2:**
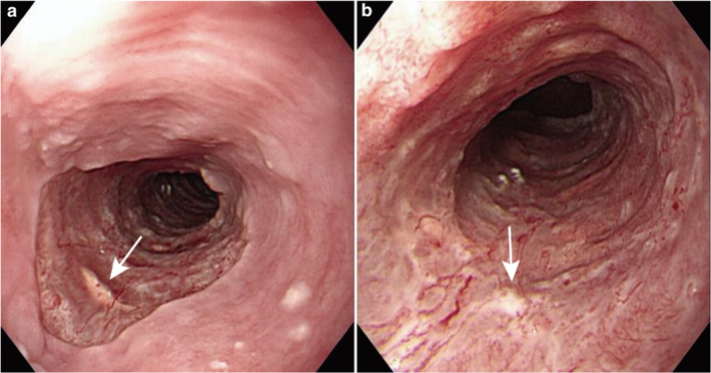
**Endoscopic view 6 days after endoscopic submucosal dissection.** Some injected triamcinolone is evident in the ulcer (white arrows). The right picture **(a)** is a magnification of the left picture **(b)**.

**Figure 3:**
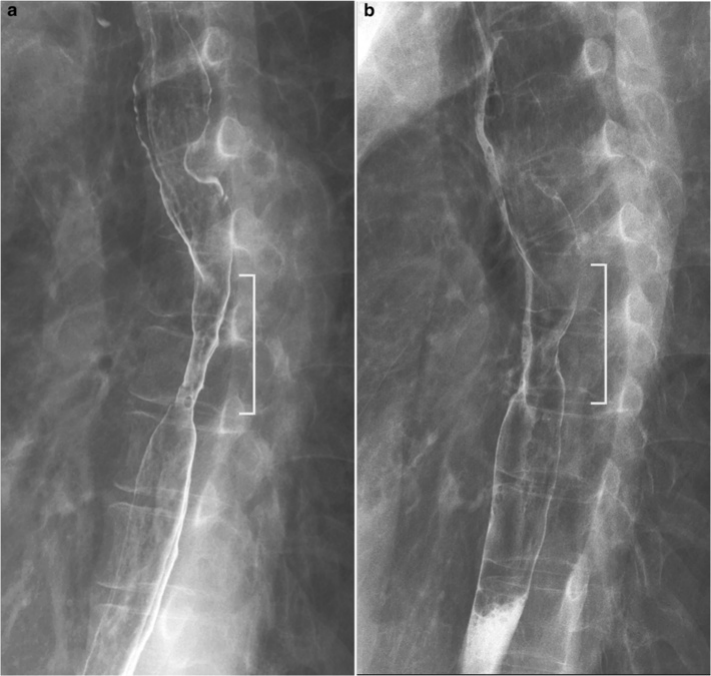
**Esophageal stenosis assessed by esophagography.** The white lines indicate the stricture caused by resection. The stricture had substantially improved 1 month later. The left esophagogram **(a)** was taken 2 months after endoscopic submucosal dissection (ESD); the right esophagogram **(b)** was taken 3 months after ESD.

**Figure 4:**
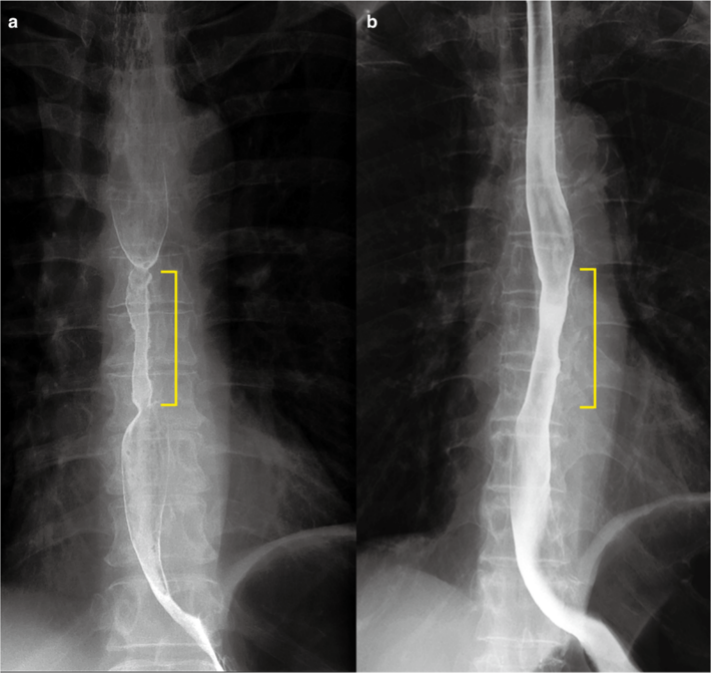
**Barium esophagography 1 month after endoscopic submucosal dissection.** The yellow lines indicate the narrow lumens owing to resection. **a**. Patient allocated to the control group, who developed a severe esophageal stricture that required 13 sessions of dilatation therapy. **b**. Patient allocated to the treatment group, who did not require dilatation.

**Figure 5:**
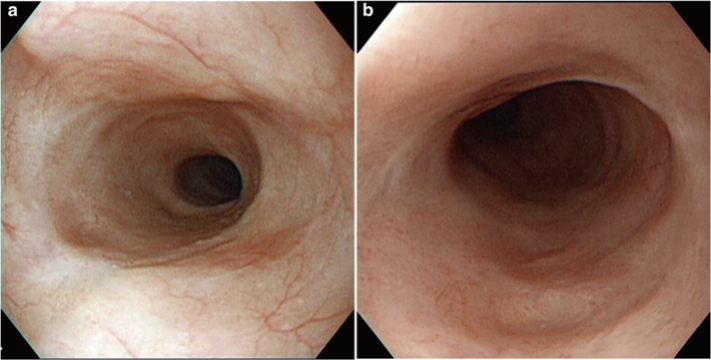
**Typical endoscopic view 1 month after endoscopic submucosal dissection.** Severe stenosis of the esophagus did not develop. The right picture **(a)** is a magnification of the left picture **(b)**.

Esophagogastroduodenoscopy (EGD) was performed to assess for stenosis, bleeding or perforation at the injected sites 6 days after treatment (Figure 2). Barium contrast esophagography was performed in patients who complained of dysphagia, or 4 weeks after the last EGD if patients were asymptomatic, to quantitatively assess stenosis (Figure 3). Esophageal stenosis was defined as an esophageal diameter <11 mm, rather than the inability to pass the gastroscope (which had a diameter of 9.8–11.0 mm) or inability to achieve or maintain a diameter of 14 mm despite dilatation every 2–4 weeks [5,16]. The luminal diameter was estimated by measurement of the minimum diameter of the stricture on esophagography (for examples, see Figures 3 and 4). Dilatation therapy was performed every 1–4 weeks as previously described [3]. Briefly, dilatation was performed in the outpatient department under fluoroscopic guidance using a Maloney (Medovations, Milwaukee, WI) or Savary (Wilson Cook Medical, Winston-Salem, NC) wire-guided dilator [17]. Dilatation therapy was considered successful when patients did not report any symptoms of dysphagia without having needed dilatation in the previous 4 weeks (Figure 5).

### Statistical analysis

The incidence of esophageal stenoses and the frequency of dilatation sessions required were compared in the treatment and control groups. Independent *t* tests were used to compare age, resection size and procedure time. The primary study endpoint was the frequency of stricture requiring endoscopic dilatation for esophageal stenosis after ESD. A surrogate primary endpoint, the number of dilatation sessions required, was subsequently included in the analysis because the primary endpoint did not reach statistical significance. Secondary endpoints included the frequency of complications that occurred as a consequence of either local steroid injection or endoscopic dilatation, the minimum diameter of the stenotic area and the duration of the course of dilatation treatments (Tables 3 and 4). The number of patients to be enrolled was determined in advance using a power calculation for two-sample proportions test based on expected bougienage rates of 13% with and 60% without steroid injection, informed by previous reports of esophageal peptic stricture rates [13], with an α error of 0.05 (two-tailed) and a β error of 0.2. Consequently, the number of patients required in each group was calculated as 16 using R statistical software [18]. *Post hoc* analysis was also undertaken to compare the group of patients with whole circumferential mucosal defects (WCMD) with the group of those with lesions that involved less than the whole circumference (non-WCMD, NWCMD; Table 4), and a further analysis of the characteristics of patients with WCMDs was made (Table 5). All other statistical analyses were performed using IBM SPSS Statistics version 21 (IBM Japan, Tokyo, Japan). Chi-square tests were used to compare nominal and ordinal variables, with the exact *P* value based on Pearson’s statistics or the Monte Carlo method applied as appropriate. We used *t* tests or Mann–Whitney tests for ratio scale variables, and for all tests a two-tailed *P* value of <0.05 was considered statistically significant.

**Table 3:** **Characteristics of dilatation procedures undertaken in the treatment and control groups**

**Treatment** ***vs.*** **Control**	**Treatment group**	**Control group**	**p-value**
	**n = 16**	**n = 16**	
**Frequency of stricture, n (%)**	10 (62.5)	14 (87.5)	0.22
**Required dilatation sessions, n**	6.1 ± 6.2	12.5 ± 10.1	0.038
**mean ± SD (range)**	(0–17)	(0–40)	
**Perforation by dilatations, n per session (%)**	1/97 (1.0)	1/200 (0.5)	0.55
**Minimum diameter of strictured region, mm mean ± SD (range)**	11.0 ± 4.6 (5.4-21.8)	7.1 ± 2.9 (5.1-12.8)	0.008
**Duration of dilatation therapy, months mean ± SD (range)**	3.5 ± 4.0 (0–13)	6.1 ± 5.0 (0–20)	0.11

Abbreviation: SD, standard deviation.

**Table 4:** ***Post hoc*** **analysis of characteristics of dilatation procedures undertaken in those with mucosal defects involving the whole circumference or less than the whole circumference of the esophagus**

**WCMD** ***vs.*** **NWCMD**	**WCMD group**	**NWCMD group**	**p-value**
	**n = 10**	**n = 22**	
**Frequency of stricture, n (%)**	10 (100)	14 (63.6)	0.035
**Required dilatation sessions, n mean ± SD (range)**	16.3 ± 9.7(6–40)	6.1 ± 6.4 (0–20)	0.013
**Perforation by dilatations, n per session (%)**	1/163 (0.6)	1/134 (0.7)	0.93
**Minimum diameter of stenotic region, mm mean ± SD (range)**	7.2 ± 2.6(5.1-12.7)	9.9 ± 4.7(5.3-21.8)	0.10
**Duration of dilatations, months mean ± SD (range)**	8.1 ± 5.2(2–20)	3.3 ± 3.5(0–20)	0.047

Abbreviations: WCMD, whole circumferential mucosal defect; NWCMD, non-WCMD; SD, standard deviation.

**Table 5:** **Subgroup analysis of patients with mucosal defects involving the whole esophageal circumference**

**Treatment** ***vs.*** **Control in the WCMD**	**Treatment subgroup**	**Control subgroup**	**p-value**
	**n = 5**	**n = 5**	
**Frequency of stricture, n (%)**	5 (100)	5 (100)	0.99
**Required dilatation sessions, n mean ± SD (range)**	10.4 ± 3.5(6–15)	22.2 ± 10.6 (13–40)	0.046
**Perforation by dilatations, n per session (%)**	0/52 (0)	1/111 (0.9)	0.99
**Minimum diameter of stenotic region, mm mean ± SD (range)**	7.7 ± 1.7 (5.4-9.6)	6.7 ± 3.4 (5.1-12.7)	0.58
**Duration for dilatations, month mean ± SD (range)**	6.2 ± 4.4 (2–13)	10.0 ± 5.7 (6–20)	0.27

Abbreviations: WCMD, whole circumferential mucosal defect; NWCMD, non-WCMD; SD, standard deviation.

## Results

**Figure 6:**
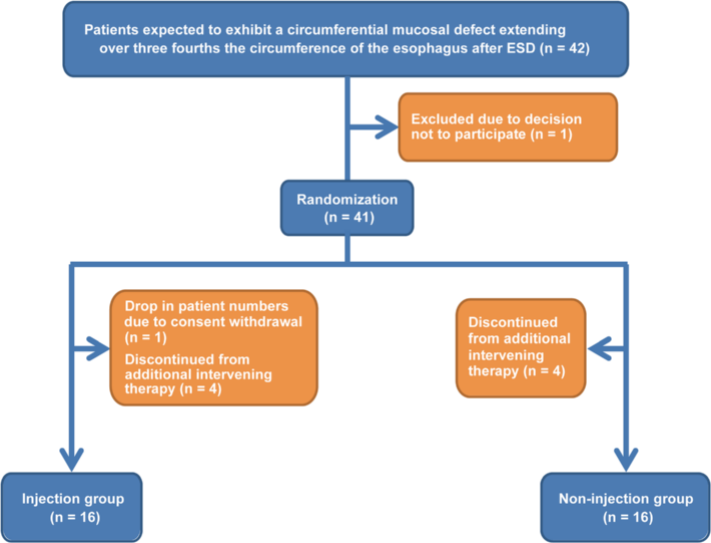
**Study flow chart.** Patients with an expected circumferential mucosal defect involving ≥75% of the circumference of the esophagus after ESD were eligible. Patients were excluded if they had received additional adjuvant treatments, such as surgery or chemoradiation therapy, or if they were not adequately followed-up.

Recruitment began in February 2010 and the last follow-up was in October 2011. The trial ended because data were considered complete. During the study period, 209 patients with 256 lesions underwent ESD in our hospital, 42 of whom (20.1%) were enrolled in the study because they were expected to have mucosal defects extending over three-quarters of the esophageal circumference due to the ESD. Since one of these 42 patients declined to participate, in total 41 were enrolled and randomized. 21 were allocated to the injection (treatment) group whereas 20 were allocated to the non-injection (control) group. However, after ESD, nine patients were excluded from the study: one whose follow-up was inadequate and eight who received additional therapy. Of the latter eight patients, seven had submucosal invasion that exceeded 200 μm and one had lymphatic invasion despite a depth of invasion of only 180 μm. Ultimately, 16 patients were allocated to each group (Figure 6).

### Comparison of treatment and control groups

Patients’ baseline demographic and clinical characteristics (Table 1) and those of the ESD procedures (Table 2) were not significantly different between the groups. No patient experienced perforation caused by steroid injection or any other side effects of steroids. The frequency of stricture was not significantly different between the treatment (n = 10, 62.5%) and control (n = 14, 87.5%) groups (*P* = 0.22, Table 3). The mean number of sessions of dilatation therapy was significantly lower in the treatment than in the control group (6.1 sessions [95% confidence interval, CI 2.8–9.4 sessions] *versus* 12.5 sessions [95% CI 7.1–17.9 sessions]; *P* = 0.04). The perforation rate caused by dilatation procedures was 1.0% (one out of 97 sessions) in the steroid injection group and 0.5% (one out of 200 sessions) in the control group. The mean minimum diameter of stenotic lumens just before dilatation therapy was greater in the treatment group than controls (11.0 mm [95% CI 8.5–13.4 mm] *versus* 7.1 mm [95% CI 5.5–8.6], *P* = 0.01; Figure 4). The duration of dilatation therapy was 3.5 months in the treatment group and 6.1 months in the control group, but the difference was not statistically significant (*P* = 0.11, Table 3).

### Post hoc comparison between patients with whole and non-whole circumferential mucosal defects

There were no significant differences in the baseline demographic, clinical or ESD characteristics of the 10 patients with WCMDs compared with the remaining 22 with NWCMDs (data not shown). The incidence of stricture was significantly more frequent (100% *versus* 63.6%, *P* = 0.035) and the mean number of dilatation therapy sessions required was significantly more (16.3 *versus* 6.1 sessions, *P* = 0.013) in those with WCMD lesions. The perforation rate caused by dilatation procedures was similar: 0.6% (one out of 163 sessions) in the WCMD group compared with 0.7% (one out of 134 sessions) in the NWCMD group. The mean minimum diameter of stenotic lumens immediately before dilatation therapy was smaller in the WCMD group (7.2 *versus* 9.9 mm in the NWCMD group) but the difference was not significant (*P* = 0.10). The mean duration of dilatation therapy was significantly longer (8.1 *versus* 3.3 months, *P* = 0.047) in the WCMD group (Table 4).

### Subgroup analysis of the whole circumference mucosal defect group

Comparisons of patients in the WCMD subgroup treated with steroid (n = 5) and those treated conventionally (n = 5) revealed no significant differences in baseline demographic, clinical or ESD characteristics (data not shown). The only treatment-related factor that differed significantly between the groups was the mean number of dilatation therapy sessions required, which was lower in those treated with steroids compared with controls (10.4 sessions [95% CI 6.0–14.8 sessions] *versus* 22.2 sessions [95% CI 9.0–35.4 sessions], respectively, *P* <0.05; Table 3).

## Discussion

Strictures have been observed to develop when the mucosal defect extends beyond three-quarters of the esophageal circumference in 68–92% of patients [2,5,9]. Because of the recent trend of treating larger lesions by extensive ESD, the number of patients with post-ESD strictures is increasing. These strictures interfere with subsequent management and impair quality of life. We therefore designed a randomized, controlled trial to examine the efficacy of endoscopic triamcinolone injection in preventing esophageal stenoses after extensive ESD, including patients with mucosal defects encompassing the entire circumference of the esophagus.

We found that endoscopic triamcinolone injection did not reduce the frequency of stricture formation, but reduced the mean number of dilatation sessions per patient from 12.5 to 6.1, suggesting that steroid injection may partially relieve esophageal stenoses. No steroid-related adverse events were observed, and the perforation rate during dilatation procedures was similar in the treated and control groups (1.0% *versus* 0.5%, respectively). These results suggest that a single prophylactic dose of steroid administered after ESD is safe and well tolerated. The mean minimum diameter of stenotic lumens immediately before the first dilatation treatment was significantly greater in the treated group than controls (11.0 *versus* 7.1 mm, respectively). The differences observed in duration of dilatation therapy were not statistically significant. Our trial is the first to demonstrate the partial but significant prophylactic effect of steroid injection on stricture formation in this clinical setting.

Hanaoka and colleagues previously stated that a randomized controlled trial comparing a single injection of steroid at the time of dilatation therapy may not be ethically acceptable, as the efficacy of steroid injection therapy is well recognized [10]. Furthermore, they stated that it would be better justified for a future controlled trial to compare multiple steroid injections with systemic steroids or a different steroid injection regime. However, previous studies – including theirs – had excluded patients with circumferential defects from their trial, as these patients are known to develop extremely severe strictures [8,19]. Moreover, patients with WCMDs have been reported to require as many as 32 dilatation sessions [19], highlighting the challenges faced by clinicians in these cases. Although we are encountering more patients with circumferential mucosal defects in our clinical practice, the best way of managing them has not been determined. The results from our *post hoc* analysis confirm that patients with WCMDs are more likely to develop strictures, require more dilatation sessions and longer duration of treatment, but that they benefited most from a single prophylactic steroid injection after ESD. In patients with WCMDs and esophageal diameters of approximately 7 mm, prophylactic steroid treatment almost halved the number of dilatation sessions needed and the overall duration of treatment. This in itself is a clinically important finding, not least because a reduced incidence of esophageal perforation would be likely to reduce morbidity and mortality. As it is well recognized that patients are at a lower risk of esophageal perforation if they undergo fewer dilatation treatments [3], prophylactic steroid injection might also improve patient safety, although as perforation rates are so low a large surveillance study would be needed to detect a difference.

Our findings also concur with those of previous studies, which showed that patients with smaller mucosal defects also benefited from endoscopic steroid injections to prevent post-ESD strictures. Our study may not have been adequately powered to detect these smaller – but nonetheless clinically relevant – differences in the frequency of stricture formation. Our results should be further confirmed by a large well-powered randomized controlled trial, which should also examine whether multiple steroid injections, administered during dilatation treatments, might benefit those patients that go on to develop esophageal stenoses.

A course of oral prednisolone has been reported to be an effective means of preventing strictures [8]. Endoscopic steroid injection is preferable to oral prednisolone in patients with diabetes mellitus, those who experience immediate or delayed adverse events after ESD, or those who require additional treatment for submucosal invasion. The systemic side effects of a locally injected steroid would likely be negligible compared with those of a systemic steroid. Histopathological analysis of excised ESD specimens showed that seven patients had tumors invading the submucosa beyond 200 μm and thus required additional therapy. Oral prednisolone would likely increase the risks associated with surgery, making it an obstacle to immediate surgical intervention. It has previously been reported that oral steroid therapy led to only three patients with WCMDs requiring a mean 0.7 sessions of dilatation therapy [8]. Thus, it remains unclear whether locally or systemically administered steroid is superior for patients with WCMDs. A head-to-head comparative study may establish a standard prophylaxis for esophageal stenosis caused by ESD.

The estimated sample size of 16 patients per group was determined by a power calculation based on expected stricture rates of 13% and 60% with and without steroid injection, respectively, informed by previously published data on esophageal peptic strictures [13]. These assumptions are now supported by another study [10], which showed that the proportion of strictures caused by ESD was 10% in steroid-treated patients and 66% in an historical control group. Nevertheless, our study might have been inadequately powered, as the stricture rates were 62.5% in the treatment group and 87.5% in the control group, a likely consequence of including 10 patients (31.3%) with circumferential mucosal defects. Furthermore, the mean numbers of dilatation sessions required in our study were higher than in previous reports [10], again likely a consequence of the relatively high proportion of patients with circumferential lesions. The patients in our study with NWCMDs underwent a similar mean number of dilatation sessions to a broadly comparable group of patients in a previous report [9]. Our study has other limitations. First, it was an open-label design conducted by a single endoscopy specialist in a single specialist center. Second, concealment was based only on pseudo-randomization. Finally, the primary outcome measure was not significantly different between the groups, and some of our conclusions of therapeutic benefit are based on *post hoc* or subgroup analysis.

## Conclusion

In summary, prophylactic endoscopic steroid injection can relieve the severity of esophageal stenoses following extensive ESD. Future studies should attempt to optimize steroid injection therapy to establish the best means of preventing stricture formation in patients at risk of developing esophageal stenosis. As it is well recognized that patients are at a lower risk of esophageal perforation if they undergo fewer dilatation treatments [3], prophylactic steroid injection might also improve patient safety, although as perforation rates are so low a large surveillance study would be needed to detect a difference.
